# Conventional versus real‐time quantitative PCR for rare species detection

**DOI:** 10.1002/ece3.4636

**Published:** 2018-11-14

**Authors:** Zhiqiang Xia, Mattias L. Johansson, Yangchun Gao, Lei Zhang, Gordon Douglas Haffner, Hugh J. MacIsaac, Aibin Zhan

**Affiliations:** ^1^ Great Lakes Institute for Environmental Research University of Windsor Windsor Ontario Canada; ^2^ International S&T Collaborative Base for Water Environment Monitoring and Simulation in Three Gorges Reservoir Region Chongqing China; ^3^ Research Center for Eco‐Environmental Sciences Chinese Academy of Sciences Beijing China; ^4^ Department of Biology University of North Georgia Oakwood Georgia; ^5^ University of Chinese Academy of Sciences Beijing China; ^6^ College of Resources and Environment Southwest University Chongqing China; ^7^ School of Ecology and Environmental Science Yunnan University Kunming China

**Keywords:** eDNA, environmental DNA, false negative, golden mussel, qPCR, rare species

## Abstract

Detection of species in nature at very low abundance requires innovative methods. Conventional PCR (cPCR) and real‐time quantitative PCR (qPCR) are two widely used approaches employed in environmental DNA (eDNA) detection, though lack of a comprehensive comparison of them impedes method selection. Here we test detection capacity and false negative rate of both approaches using samples with different expected complexities. We compared cPCR and qPCR to detect invasive, biofouling golden mussels (*Limnoperna fortunei*), in samples from laboratory aquaria and irrigation channels where this mussel was known to occur in central China. Where applicable, the limit of detection (LoD), limit of quantification (LoQ), detection rate, and false negative rate of each PCR method were tested. Quantitative PCR achieved a lower LoD than cPCR (1 × 10^−7^ vs. 10^−6^ ng/μl) and had a higher detection rate for both laboratory (100% vs. 87.9%) and field (68.6% vs. 47.1%) samples. Field water samples could only be quantified at a higher concentration than laboratory aquaria and total genomic DNA, indicating inhibition with environmental samples. The false negative rate was inversely related to the number of sample replicates. Target eDNA concentration was negatively related to distance from sampling sites to the water (and animal) source. Detection capacity difference between cPCR and qPCR for genomic DNA and laboratory aquaria can be translated to field water samples, and the latter should be prioritized in rare species detection. Field environmental samples may involve more complexities—such as inhibitors—than laboratory aquaria samples, requiring more target DNA. Extensive sampling is critical in field applications using either approach to reduce false negatives.

## 1 INTRODUCTION

Accurately detecting rare species—such as newly introduced nonindigenous species (NIS) or endangered native species—is critical for both conservation and management. Imperfect detection through either false positive or false negative results impedes these efforts, particularly with respect to rapid response to NIS incursions (Zhan & MacIsaac, 2015). However, detecting these species is challenging either because of their small population size and/or geographically‐constrained distribution (Branstrator, Beranek, Brown, Hembre, & Engstrom, 2017; Robertson et al., 2017; Simberloff et al., 2013; vander Zanden, 2010).

Environmental DNA (eDNA) refers to DNA released by organisms into their environment and is distributed where species currently or previously existed or where it has been advected from these sources. eDNA can be extracted from bulk environmental samples and thus can be targeted and amplified using properly designed PCR primers (see Taberlet, Coissac, Hajibabaei, & Rieseberg, 2012). eDNA is particularly useful for fast, sensitive and accurate species detection and discrimination at low abundance (Bohmann et al., 2014; Jerde, Mahon, Chadderton, & Lodge, 2011; Rees, Maddison, Middleditch, Patmore, & Gough, 2014; Zhan & MacIsaac, 2015). This feature has resulted in deployment of eDNA‐based methods as a sensitive detection tool for a broad variety of aquatic species (e.g. Jerde et al., 2011; Boothroyd, Mandrak, Fox, & Wilson, 2016; Agersnap et al., 2017; Jackson, Myrholm, Shaaw, & Ramsfield, 2017; Torresdal, Farrell, & Goldberg, 2017; Voros, Marton, Schmidt, Gal, & Jelic, 2017). Despite this, eDNA‐based techniques are immature, and technical limitations must be considered when planning to employ these tools (see Wilcox et al., 2013; Goldberg, Strickler, & Pilliod, 2014; Deiner, Walser, Mächler, & Altermatt, 2015; Goldberg et al., 2016).

Technical problems may complicate interpretation of eDNA results (Rees et al., 2014). For example, cross‐contamination during sample collection, transport, or laboratory preparation may cause false positive results (i.e. target NIS is absent but DNA is detected in samples; Goldberg et al., 2016), while false negatives (i.e. target NIS is present but DNA is not detected) can occur if inhibitors are present in eDNA used as PCR templates (Jane et al., 2015) or if PCR primers have insufficient sensitivity (Wilcox et al., 2013; Xiong, Li, & Zhan, 2016). It is imperative that detection programs have a low false negative rate given that they may delay recognition of, and rapid response to, presence of NIS, or may fail to detect a target endangered species. According to Goldberg et al (2016), an eDNA‐based survey has two primary tasks: eDNA retrieval (e.g. sample collection and DNA extraction) and eDNA amplification (e.g. inhibitor removal and PCR). Many studies have focused on the former to improve detection rate (e.g. Renshaw, Olds, Jerde, Mcveigh, & Lodge, 2014; Takahara, Minamoto, & Doi, 2014; Deiner et al., 2015; Spens et al., 2016; Hinlo, Gleeson, Lintermans, & Furlan, 2017; Xia et al., 2018), while attention has rarely been paid to the latter. Given that eDNA is often found in trace amounts (Furlan, Gleeson, Hardy, & Duncan, 2016), robust PCR methods are essential to eDNA‐based studies.

At present, conventional PCR (cPCR) and real‐time quantitative PCR (qPCR) are the two major approaches used in eDNA‐based species detection. Droplet digital PCR (ddPCR) has been suggested more sensitive than both, though it currently has limited use owing to cost and operational complexity (Doi et al., 2015; Nathan, Simmons, Wegleitner, Jerde, & Mahon, 2014). A review of the literature revealed that 37% and 61% of eDNA studies employed cPCR and qPCR, respectively, for aquatic species detection (Z. Xia, unpublished). It has been suggested that qPCR, which is a quantitative or semi‐quantitative method, is the more sensitive method (Balasingham, Walter, & Heath, 2017), although cPCR is more readily available to most molecular laboratories. This wide availability lends itself to greater use in rare species detection (Ojaveer et al., 2014; Ricciardi et al., 2017; Roy et al., 2015), as it is cost‐efficient and can be very sensitive (e.g. Jerde et al., 2011). Ideally, a robust method for environmental samples should maintain sensitivity for samples obtained from different sources. Therefore, comparison of the two most widely used PCR methods for samples from different sources may assist in future method selection for rare species detection. To our knowledge, however, this has not been well explored although several studies have discussed detection probability for eDNA samples using both methods. For example, Nathan et al. (2014) quantified eDNA signals using cPCR, qPCR, and ddPCR from mesocosm aquaria and observed 100% detection of target species across all platforms; however, they did not distinguish detection power of cPCR or qPCR. In another study, Piggott (2016) observed a higher detection rate of fish from dam water samples using qPCR than cPCR, though the investigators had limited sample sources. Additional empirical evidence derived from various systems is critical to guide future method selection.

In this study, we compared cPCR with qPCR to detect a highly invasive mollusk, the golden mussel *Limnoperna fortunei,* from environmental water samples. First, we determined the limit of detection (LoD) of each PCR method under their respective optimal conditions using total genomic DNA. Subsequently, we tested water samples from both laboratory aquaria and natural irrigation channels containing target DNA and calculated false negative rate of each method while varying sample replication. Finally, we calculated quantification level of qPCR among the aforementioned samples which differed in complexity, and compared species detectability using both methods to explore performance difference.

## 2 METHODS

### 2.1 Sample collection and DNA extraction

Animals used in this study were collected from the Danjiangkou Reservoir, China (32°39′0″N, 111°41′15″E) and reared in a 60 L tank at 24°C before use. We used water samples maintained in laboratory aquaria and from the natural environment to test the two PCR methods. To prepare laboratory samples, we reared a golden mussel clump (12 adult individuals) at 24°C in a 15 L well‐aerated aquarium for 24 hr. We then removed animals from the tank and stopped aeration. The tank was left undisturbed for 12 hr before we began to collect water samples. Three 50 ml water samples were collected from the surface layer (~10 cm) of the aquarium, using separate 50 ml syringes for each replicate. We sampled at 11 time points over the course of a week, yielding 33 samples (Supporting Information Table S1).

**Figure 1:**
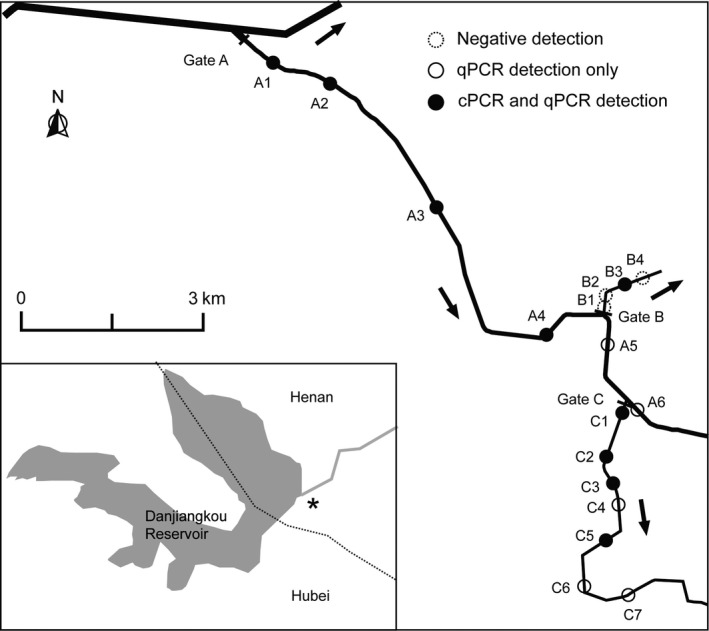
Map of sampling sites in the three irrigation channels (A, *n* = 6; B, *n* = 4; C, *n* = 7), identifying the location of each site and detection results of the golden mussel (*Limnoperna fortunei*) by both conventional PCR and quantitative PCR. Arrows indicate the direction of water flow. Three replicate samples were collected per site, and sampling was carried out from downstream to upstream. Inset indicates location of the study area (asterisk), and dotted line indicates boundary of Henan Province and Hubei Province, China

To prepare natural water samples, we sampled three irrigation channels in Dengzhou, China (Figure 1). These channels were expected to contain eDNA of the golden mussel since the species was recorded in the vicinity in a preliminary field survey. Water source in each channel was controlled by a discharge gate at its source (Figure 1). The discharge gates A and C were open while gate B was closed during sampling. Average water velocity was about 0.5 and 0.2 m/s in channels A and C, respectively, while channel B was static as the discharge gate B was completely closed. Water depth of channels A, B, and C were about 1.8, 0.4, and 0.6 m, respectively. Sample collection order was channel C, B, and then A, and always from the downstream to upstream sites. We collected three 100 ml water samples from the surface layer (~20 cm) at each site (*n* = 17), yielding a total of 51 samples. All samples were transported on ice to the laboratory within 24 hr of collection, and each was filtered onto a cellulose acetate microporous membrane filter (0.45 μm pore size). Each filter was cut in half and separately stored in a 2 ml centrifuge tube at −20°C until DNA extraction.

Total genomic DNA was extracted from fresh tissue of golden mussel using the DNeasy Blood & Tissue Kit (Qiagen). A randomly selected half‐filter for each sample was extracted using the phenol‐chloroform‐isoamyl alcohol (PCI) method of Renshaw et al. (2014). Original DNA extracts were diluted 1:10 prior to use in PCR to reduce potential influence of PCR inhibitors (McKee, Spear, & Pierson, 2015).

### 2.2 cPCR analyses

We used a species‐specific primer pair developed by Xia et al. (2018) to target a 197 bp fragment of the mitochondrial cytochrome *c* oxidase subunit I (COI) gene of the golden mussel. We ran 20 µl PCR mix following the methods detailed in Xia et al. (2018) with minor revisions: 5 µl template DNA was used in each reaction and 58°C was applied as the annealing temperature in this study. PCR products were visualized on 1.5% agarose gels using an automatic gelatin image analysis system (JiaPeng, Shanghai, China) and target bands were identified by eye. The LoD of the cPCR was tested using 10× serial dilutions of total genomic DNA with a concentration of 1.0 × 10^0^–10^−8^ ng/μl. A total of 10 replicates for each concentration was applied, and the LoD was defined as the lowest concentration returning at least one positive replicate (Agersnap et al., 2017). We Sanger‐sequenced four random positive amplicons of the field samples to confirm specificity of our primers, which was identified as species‐specific in a previous study (Xia et al., 2018).

### 2.3 qPCR analyses

We used linear regression of quantification cycle (*C*_q_) on DNA concentration (i.e. Log quantity) by amplifying the same serial dilutions of total genomic DNA mentioned above. Five replicates for each concentration were applied to construct the standard curve, and five no‐template‐controls (NTC) using double‐distilled water (ddH_2_O) were applied on the same 96‐well plate to act as negative controls. We used instrumental default parameters – 20 µl PCR mix containing 1× SYBR Green master mix (Roche Applied Science, Germany), 0.4 μM each primer, and 5.0 µl DNA template (i.e. 1:10 diluted eDNA) on a LightCycler^®^ 96 Instrument (Roche Applied Science, Germany). The thermal profile contained 60 s pre‐incubation (95°C), followed by 50 cycles of 10 s for denaturation (95°C), 20 s for annealing (62°C), and 30 s for extension (72°C), followed by 10 min for final extension. Our primer pair can successfully amplify golden mussel at an annealing temperature from 45–65°C (Xia et al., 2018), and we used 58°C for cPCR and 62°C for qPCR, respectively, as they were proven optimal in pilot experiments. A melting analysis (95°C/10 s, 65°C/60 s, 97°C/1 s) was conducted following the amplification to generate a melting curve for PCR product in each well. The LoD of qPCR was identified as the lowest concentration producing at least one positive detection out of the five replicates.

After qPCR, all melting curves were examined prior to the use of the returned *C*_q_ values by the built‐in software. Specific amplification of our target species was characterized by a peak at the correct melting temperature (Peñarrubia et al., 2016; Smith & Osborn, 2009), which was generated from amplifications of a high concentration of total genomic DNA (e.g. 1.0 ng/μl). The *C*_q_ values returned from specific amplifications were identified as valid when the corresponding melting curves were normally distributed, otherwise the *C*_q_ values were dismissed (invalid *C*_q_). To plot the standard curve, only serial dilutions of the total genomic DNA with ≥3 valid *C*_q_ values were considered. The corresponding efficiency of qPCR was calculated by the built‐in software and descriptors of the standard curve were reported following Smith and Osborn (2009).

All amplification results of water samples underwent the same procedure as the standard curve prior to the use of *C*_q_. Specifically, for those samples which returned positive amplifications but invalid *C*_q_ values (i.e. their melting curves were skewed or peaked at the NTC melting temperature), new *C*_q_ values were assigned to them according to the shape of the melting curves. The limit of quantification (LoQ) refers to the lowest concentration where the target species can be reliably quantified (Armbruster & Pry, 2008), and we defined it as the lowest concentration returning all positive replicates according to Agersnap et al. (2017). A linear regression model was applied to test the relationship between eDNA concentration (i.e. *C*_q_) in irrigation channels and the distance to water source (i.e. discharge gate).

We also tested the importance of collecting replicate samples per time‐point/site to reduce false negative results. We calculated the false negative rate when collecting between one and three replicates per time‐point/site, using the scenario with highest detection rate as a baseline. For the one‐sample scenario, each sample was considered as a replicate. Alternatively, every possible two‐sample combination was assessed in the two‐sample scenario. For both PCR methods, all laboratory and field samples that initially failed to amplify underwent a second amplification and the results of both amplification attempts were combined to calculate the detection rate. One sampling time‐point/site was considered a positive detection if any replicate tested positive.

### 2.4 Quality control

To prevent cross‐contamination during sample collection, we used new bottles for water sample collection. Two bottles filled with deionized water and transported with sampling bottles during each sampling trip served as sampling controls. In the laboratory, all nondisposable equipment (i.e. forceps, scissors, beakers, syringes, and filtration platform) involved in sample collection, filtration, and DNA extraction were treated using 10% commercial bleach for a minimum of 10 min before use to destroy residual DNA, followed by thorough rinse with deionized water to remove the bleach. Blank controls were incorporated during the process of water sample filtration, and negative controls using ddH_2_O were included in all PCRs to monitor contaminations in laboratory practice.

## 3 RESULTS

### 3.1 Limit of detection and quantification

The LoD was tested at 1 × 10^−6^ and 1 × 10^−7^ ng/μl for cPCR and qPCR, respectively (Supporting Information Table S2), indicating higher sensitivity of the latter method. For qPCR, one of five NTC replicates exhibited amplification signals (*C*_q_: 39.16) with a melting temperature of 77–78°C (Supporting Information Figure S1 lower). All high concentrations (i.e. >1 × 10^−5 ^ng/μl) of genomic DNA returned valid *C*_q_ values with a melting temperature of 79–80°C (Supporting Information Figure S1 upper). eDNA at low concentrations (i.e. ≤1 × 10^−5 ^ng/μl) was partially amplified, returning either valid (i.e. positive amplifications with normally distributed melting curves), invalid (i.e. skewed melting curves or NTC amplifications), or no *C*_q_ values (i.e. no amplification signals). The standard curve (Supporting Information Figure S2) was plotted using serial dilution of 1.0 × 10^0^–10^−5^ ng/μl in which three valid *C*_q_ values were returned at 1.0 × 10^−5^ ng/μl and five valid *C*_q_ values at higher concentrations (Supporting Information Table S2). Amplification efficiency of qPCR was 98%. The LoQ of total genomic DNA of qPCR was identified as 1.0 × 10^−4^ ng/μl.

### 3.2 Detection of laboratory and field water samples

All positive amplifications of water samples (except the ones that exclusively exhibited NTC fluorescence signals) demonstrated species‐specificity. We assigned 33, 34, and 35 as *C*_q_ to those water samples which exhibited positive amplification of target species but returned skewed melting curves (Supporting Information Figure S3). These values were assigned to ensure that they were at least 3.3 fewer than those from NTC (Smith & Osborn, 2009), and to guarantee an approximately continuous distribution of sample concentrations. All sampling controls and laboratory blanks demonstrated no amplifications of the target species by either PCR method throughout this study, and four randomly sequenced samples returned correct identification of the golden mussel from the field samples.

**Figure 2:**
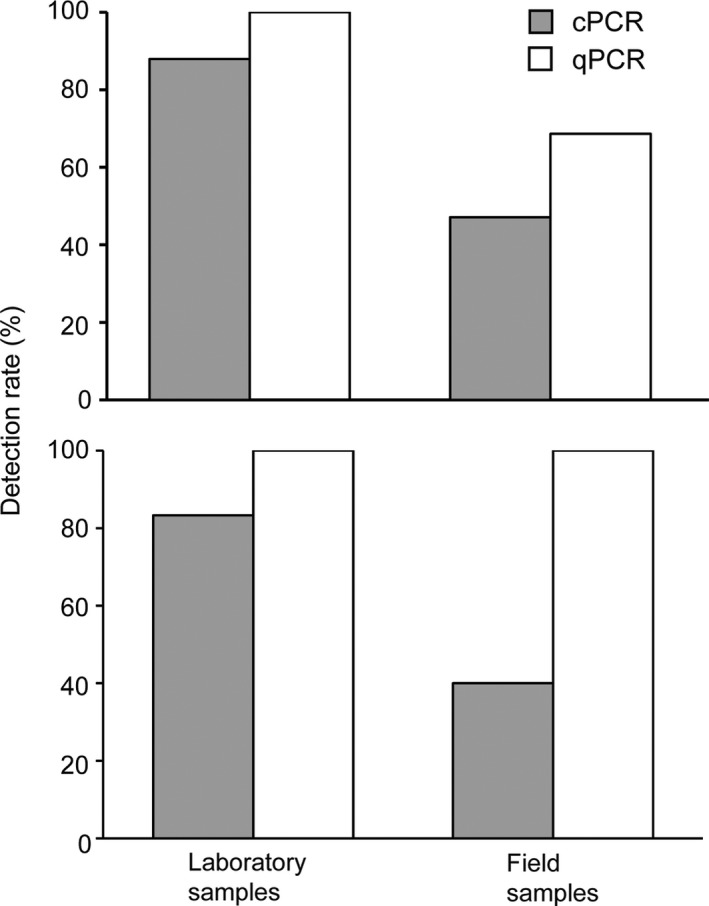
Detection rate of (upper) all replicate samples from laboratory aquaria (*n* = 33) and field (*n* = 51) and (lower) a subset of the former (*n* = 12) and latter (*n* = 15) in which quantification cycle (*C*_q_) values were assigned to samples owing to skewed melting curves

**Figure 3:**
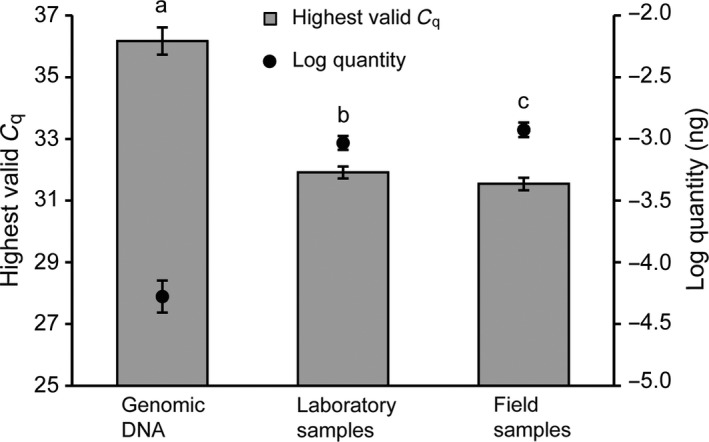
Mean (±*SD*) of three lowest quantities (solid circle) and their valid quantification cycle (*C*_q_) values (bar) of target DNA detected from total genomic DNA, laboratory samples, and field samples, respectively, using quantitative PCR. *C*_q_ refers to the number of cycles required for fluorescent signals to reach a threshold. Different letters indicate significant differences (*p* < 0.05)

Quantitative PCR achieved a higher detection rate than cPCR in both laboratory (100% vs. 87.9%) and field (68.6% vs. 47.1%) sample replicates (Figure 2 upper), resulting in five more sites detected positive in water channels (Figure 1) by the former method. For those sample replicates that were assigned *C*_q_ values, 83.3% of laboratory samples (*n* = 12) and 40% of field samples (*n* = 15) were also detected positive using cPCR (Figure 2 lower). Positive detections by cPCR were always a subset of those by qPCR. We found significant differences among quantifying total genomic DNA, laboratory aquaria, and field samples by qPCR by comparing the three lowest concentration (i.e. three highest valid *C*_q_ values) of each group (Figure 3). Specifically, total genomic DNA could be quantified to a significantly lower level (10^−4.28 ± 0.13^ ng; ANOVA, *F*_2,6_ = 218, *p < *0.001) than either laboratory (10^−3.03 ± 0.06^ ng) or field samples (10^−2.92 ± 0.06^ ng). Furthermore, laboratory samples could be quantified to a significantly lower amount than field samples (*t*_4_ = −2.273, *p = *0.043, one‐tailed).

**Figure 4:**
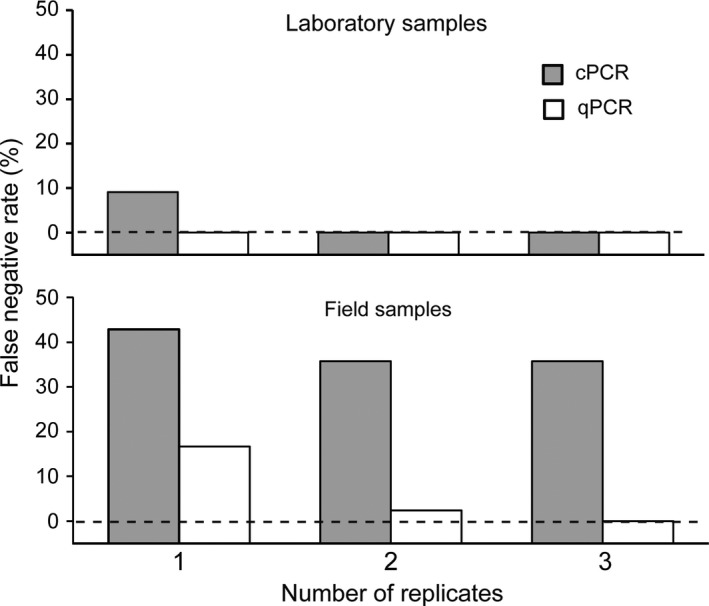
False negative rate using one, two, and three replicates in the laboratory (upper) and in the field (lower) using cPCR (grey bar) and qPCR (white bar). Dashed line indicates 100% positive detections for laboratory samples (upper) and positive detections for field samples (lower) determined by quantitative PCR when three replicates were used

**Figure 5:**
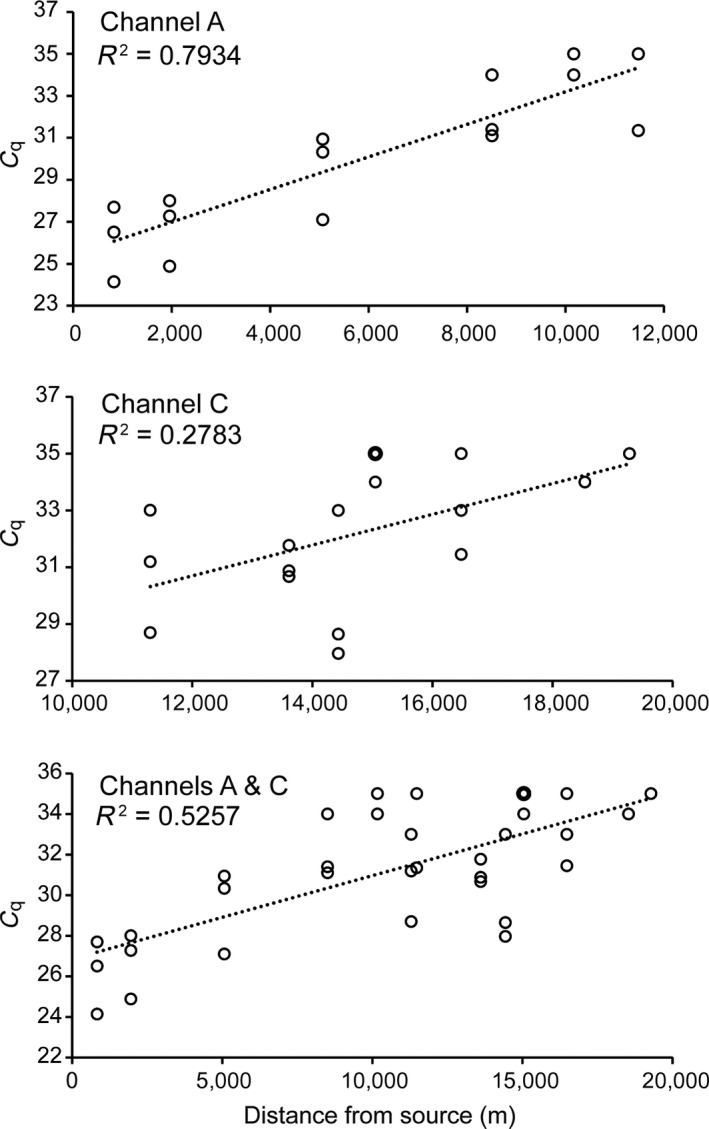
Linear regression of quantification cycle (*C*_q_) against distance to water source (gate A) for water samples collected from channel A (upper, *df* = 15, *p* < 0.001), channel C (middle, *df* = 16, *p* = 0.03), and combination (lower, *df* = 32, *p* < 0.001). Each circle indicates a replicate showing positive detection of golden mussel by quantitative PCR, and the thicker circles indicate two overlapped replicates. Note that only one or two replicates were available for some sites

False negative detections were observed using both PCR methods when only one replicate sample was collected, though this rate declined by utilizing additional sample replicates (Figure 4). Specifically, the false negative rate of cPCR decreased from 9.1% to 0%, and from 42.9% to 35.7% when sample replicates increased from one to three for laboratory and for field samples, respectively. *C*_q_ values were positively correlated with distance from the water source in channels A (Figure 5 upper, *p* < 0.001) and C (Figure 5 middle, *p = *0.03) or their combination (Figure 5 lower, *p* < 0.001), indicating a decrease in DNA concentration with distance downstream.

## 4 DISCUSSION

Conventional PCR and qPCR methods are essentially the same with respect to amplifying target fragments (Smith & Osborn, 2009). An important reason why qPCR was suggested to be more sensitive than cPCR is that different methods are utilized to detect PCR products; the former detects PCR products on‐site by measuring fluorescence in each single PCR plate well, providing higher sensitivity than the ethidium bromide‐stained, gel‐based detection under ultraviolet light used in this study. qPCR detects PCR products at the exponential stage of the PCR phase while cPCR does so at the plateau stage (Smith & Osborn, 2009), allowing the former to be less vulnerable to product degradation at high reaction cycles as reagents are exhausted. This characteristic also restrains cPCR to be conducted with fewer cycles than the former (e.g. Nathan et al., 2014) due to accumulation of artifacts (e.g. chimeras) at higher numbers of cycles (Qiu et al., 2001; Smith & Osborn, 2009). Furthermore, qPCR can exclude ambiguity of positive/negative interpretation which may cause bias in cPCR (Nathan et al., 2014). We determined a lower LoD with qPCR than cPCR (i.e. 1.0 × 10^−7^ vs. 1.0 × 10^−6^ ng/μl) which highlights an advantage of the former, while both methods exhibited 100% successful amplification at higher DNA concentrations (≥1 × 10^−4^ ng/μl). This is consistent with previous studies that conducted species detection in laboratory aquaria (e.g. Nathan et al., 2014), indicating that detection probability of cPCR and qPCR may differ only at low concentrations. To push detection limit to even lower levels, more effort is required to optimize PCR protocols or to improve primers design to reduce possible dimers. We used a 10× dilution to prepare varying total genomic DNA concentrations and only limited amplification success were observed in low concentrations (i.e. 1 × 10^−5^−10^−7^ ng/μl). In future studies, a more refined dilution series (e.g. 2×) could be used to determine a refined LoD difference of both PCR methods. It should be acknowledged that formulating more sensitive PCR protocols based on the cPCR detection mechanism is possible. For instance, nested PCR which is widely used in diagnostic laboratories, can offer almost double the number of cycles of amplification by using nested duplex primer pairs (e.g. Sotlar et al., 2004), and can be comparable with qPCR regarding detection sensitivity under some circumstances (e.g. Cullen, Lees, Toth, & Duncan, 2001). However, wide application of it in rare species detection from environmental samples may be challenged because of its operational complexity and time required.

Quantitative PCR achieved a higher detection rate for water samples than cPCR (Figure 2 upper), consistent with observations in previous studies (e.g. Piggott, 2016), reflecting the higher sensitivity (or lower LoD) of the former. In addition to LoD difference, PCR inhibitors, which occur widely in environmental samples (McKee et al., 2015), may also contribute to detection rate difference between the two methods. PCR inhibitors such as humic acid or nontarget species DNA may impact the final quality of eDNA (Pedersen et al., 2015; Wilson, 1997), affecting PCR efficiency. Relative to total genomic DNA, DNA in environmental samples may have a more uncertain fate owing to various factors such as season, UV, pH, temperature, substrate type, and downstream transport (Buxton, Groombridge, & Griffiths, 2017; Jane et al., 2015; Strickler, Fremier, & Goldberg, 2015), and will likely contain higher amounts of impurities that inhibit amplification and result in lower PCR efficiency (Pedersen et al., 2015). This view is consistent with the finding that target DNA can be quantified (i.e. valid *C*_q_ values returned) to a lower level for total genomic DNA than for laboratory or field samples using qPCR (Figure 3). We expect that both cPCR and qPCR may suffer from inhibition in the same manner, however, we observed a greater detection rate difference between methods for all sample replicates from field than from laboratory samples (21.5% vs. 12.1%; Figure 2 upper). Furthermore, for the subset samples that were assigned *C*_q_ values due to skewed melting curves, a greater detection rate difference (60% vs. 16.7%) was observed in field samples (Figure 2 lower). This additional evidence is consistent with the view that sample complexity may affect PCR success and that qPCR is more tolerant than cPCR to inhibitors owing to its more sensitive detection mechanisms (Smith & Osborn, 2009). This observation is consistent with Doi et al. (2015), who studied qPCR and droplet digital PCR. It should be acknowledged that the master mix used in each PCR method may also affect detection efficiency (Jane et al., 2015) and contribute to detection differences. We tried to reduce inhibitors by using diluted eDNA extracts (Bustin et al., 2009; McKee et al., 2015), though we were unable to identify and quantify inhibitors of different samples in this study. Future studies are needed to assess impact of eDNA complexity (or presence of inhibitors) on detection performance for different PCR methods (Dingle, Sedlak, Cook, & Jerome, 2013; Wilson, 1997), and to explore more efficient ways to eliminate inhibitors (e.g. environmental mix) without dilution as it may reduce target DNA to undetectable levels and cause false negatives (Buxton et al., 2017).

A critical concern in the application of eDNA methods to detect rare species is occurrence of false negatives (Ficetola et al., 2015). We observed a higher detection rate of qPCR than cPCR, suggesting that the former should be embraced in rare species management since it was more sensitive and less prone to false negatives. A number of avenues exist to reduce false negatives including judicious deployment of replicates in field sampling and in the laboratory (Pedersen et al., 2015; Piaggio et al., 2014) and the use of highly sensitive PCR methods (Doi et al., 2015; Xia et al., 2018). We found that the false negative rate was inversely related to the number of replicates used per time‐point/site (Figure 4). This finding is consistent with other studies (Ficetola et al., 2015; Furlan et al., 2016) and highlights the importance of enhanced sampling effort to reduce false negatives. In this study, one replicate was sufficient to demonstrate the species presence/absence in laboratory samples, while three replicates were required for field samples (Figure 4). We used three replicates as our baseline to calculate false negative rate, which reflected the true rate of samples from laboratory aquaria and channels A and C as they were detected at 100% of sites. However, estimation of false negative rate for samples from channel B was difficult as both methods detected at only a single site. Given that many factors may cause failed detection (see Darling & Mahon, 2011), estimation of false negative rate is difficult when detection rate with a baseline is <100%.

We found that eDNA concentration in channels A and C decreased with distance from the source (Figure 1, gate A), consistent with other studies in flowing systems (Balasingham et al., 2017; Pilliod, Goldberg, Arkle, & Waits, 2013; Shogren et al., 2017; Thomsen et al., 2012). Contributors to this distribution pattern in lotic systems include facilitated degradation (Thomsen et al., 2012), dilution (Balasingham et al., 2017), and particle settlement (Jane et al., 2015). Only one‐sample replicate was tested positive at very downstream sites (i.e. C7 & C8, Figures 1 and 5), indicating limited detection probability of our method. We observed higher concentrations at sites C1–C3 than A5–A6 (Figures 1 and 5) even though the former sites are located downstream of the latter. Two factors may explain this pattern. First, water flow through gate C (Figure 1) may have facilitated particle resuspension, adding eDNA to the surface layer. Secondly, water entering channel C through gate C (Figure 1) was from the deeper—and possibly eDNA enriched—layer in channel A, than in the surface layer at sites A5–A6. Regression of *C*_q_ against transport distance in channel C explained less variance (i.e. lower *R* ^2^) than in channel A (Figure 5). This is likely because channel C is more vulnerable to human disturbance (e.g. irrigation drainage) and has higher structural heterogeneity within the channel (e.g. bottom plant growth) than channel A, as the former is smaller and shallower. However, the declining trend of eDNA with flow distance was significant when channels A and C were combined (Figure 5, lower), indicating that eDNA downstream transport may depend on water flow and spatial scale (Deiner & Altermatt, 2014; Shogren et al., 2017).

## AUTHOR CONTRIBUTIONS

Z.X., H.J.M, and A.Z. conceived the study. Z.X. performed the experiment and Y.G. assisted in samples collection and DNA extraction. Z.X. drafted the manuscript and H.J.M, M.L.J,. and A.Z. edited the manuscript and all authors reviewed the final draft of manuscript.

## DATA AVAILABILITY

Additional supporting information is available online for this article.
