# Supplementary material for: Conventional versus real‐time quantitative PCR for rare species detection
Source: Ecol Evol. 2018 Nov 14;8(23):11799–807. doi: 10.1002/ece3.4636 (PMC6303721; doi:10.1002/ece3.4636)

**Supporting information**

**Table S1** Sampling scheme of laboratory water samples. Time interval was 12 hours for days with two sampling points, and 24 hours for days with one sampling point.

| Day | 1 | 2 | 3 | 4 | 5 | 6 | 7 |
| --- | --- | --- | --- | --- | --- | --- | --- |
| # of sampling points | 2 | 2 | 2 | 2 | 1 | 1 | 1 |

**Table S2** Determination of limit of detection (LoD) of cPCR (conventional PCR, upper) and qPCR (quantitative PCR, lower). Note that cPCR had 10 replicates each concentration, and qPCR had five replicates each concentration.

| Concentration (1.0×) | 10^0^ | 10^-1^ | 10^-2^ | 10^-3^ | 10^-4^ | 10^-5^ | 10^-6^ | 10^-7^ | 10^-8^ |
| --- | --- | --- | --- | --- | --- | --- | --- | --- | --- |
| # of positive amplification (cPCR) | 10 | 10 | 10 | 10 | 10 | 6 | 1 | 0 | 0 |
| Concentration (1.0×) | 10^0^ | 10^-1^ | 10^-2^ | 10^-3^ | 10^-4^ | 10^-5^ | 10^-6^ | 10^-7^ | 10^-8^ |
| # of positive amplification (qPCR) | 5 | 5 | 5 | 5 | 5 | 3 | 0 | 1 | 0 |

**Table S3** Detection rates of target species by cPCR or qPCR in three surveyed channels. A sampling site was identified as positive detection if any of three replicate samples detected positive.

| Method | Channel A | Channel B | Channel C |
| --- | --- | --- | --- |
| cPCR | 4/6 | 1/4 | 4/7 |
| qPCR | 6/6 | 1/4 | 7/7 |

**Fig. S1** Melting curve of qPCR amplifying high concentration of genomic DNA (upper) and No-Template-Control (NTC, lower), showing melting temperature 79 ~ 80 ºC for golden mussel and 77 ~ 78 ºC for NTC respectively.


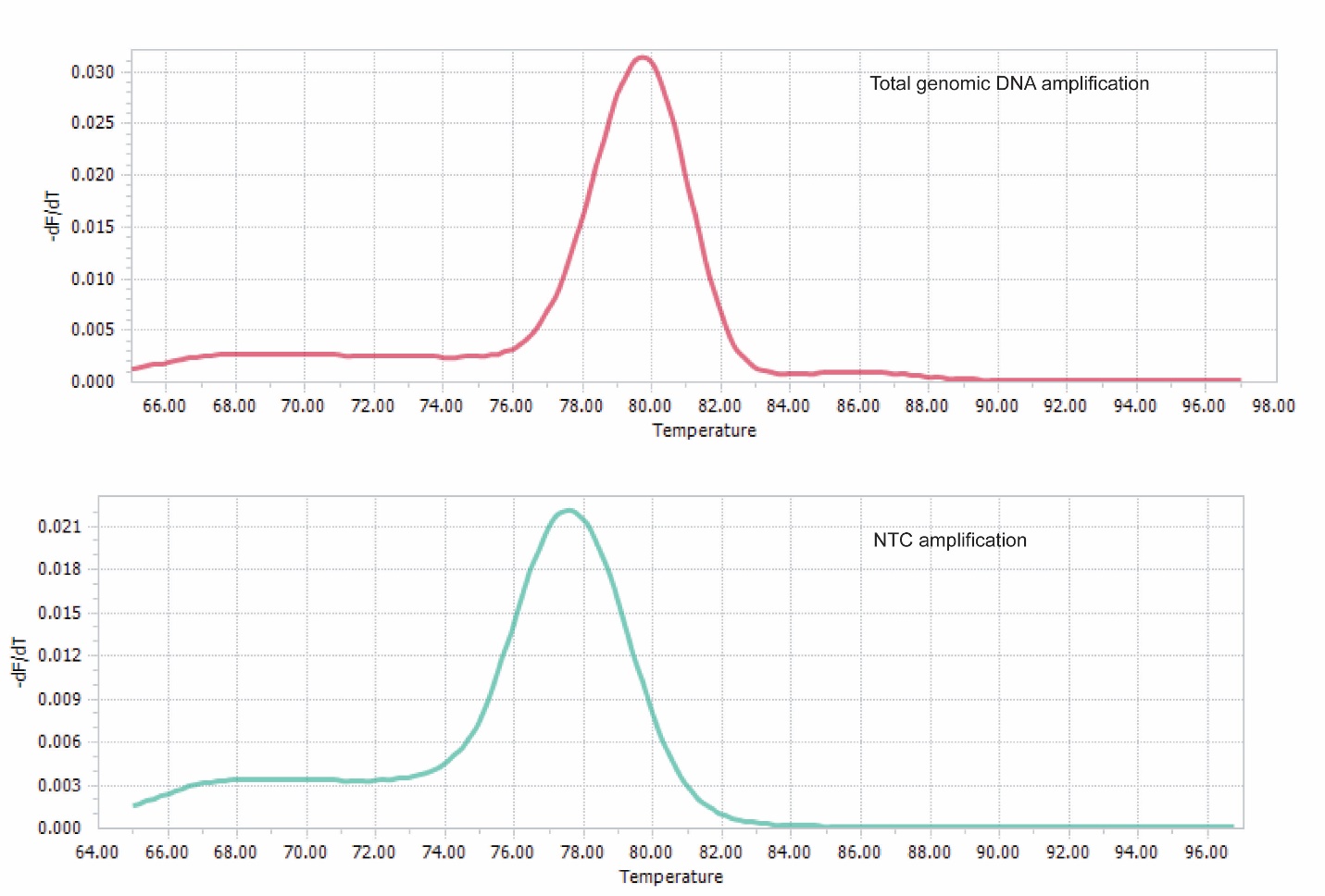


**Fig. S2** Standard curve of qPCR, demonstrating the linear regression relationship between Cq and quantity of total genomic DNA (Log Quantity) used in each reaction. Each point is the mean (± SD) of five replicates, except for the lowest concentration which only had three valid Cq values.


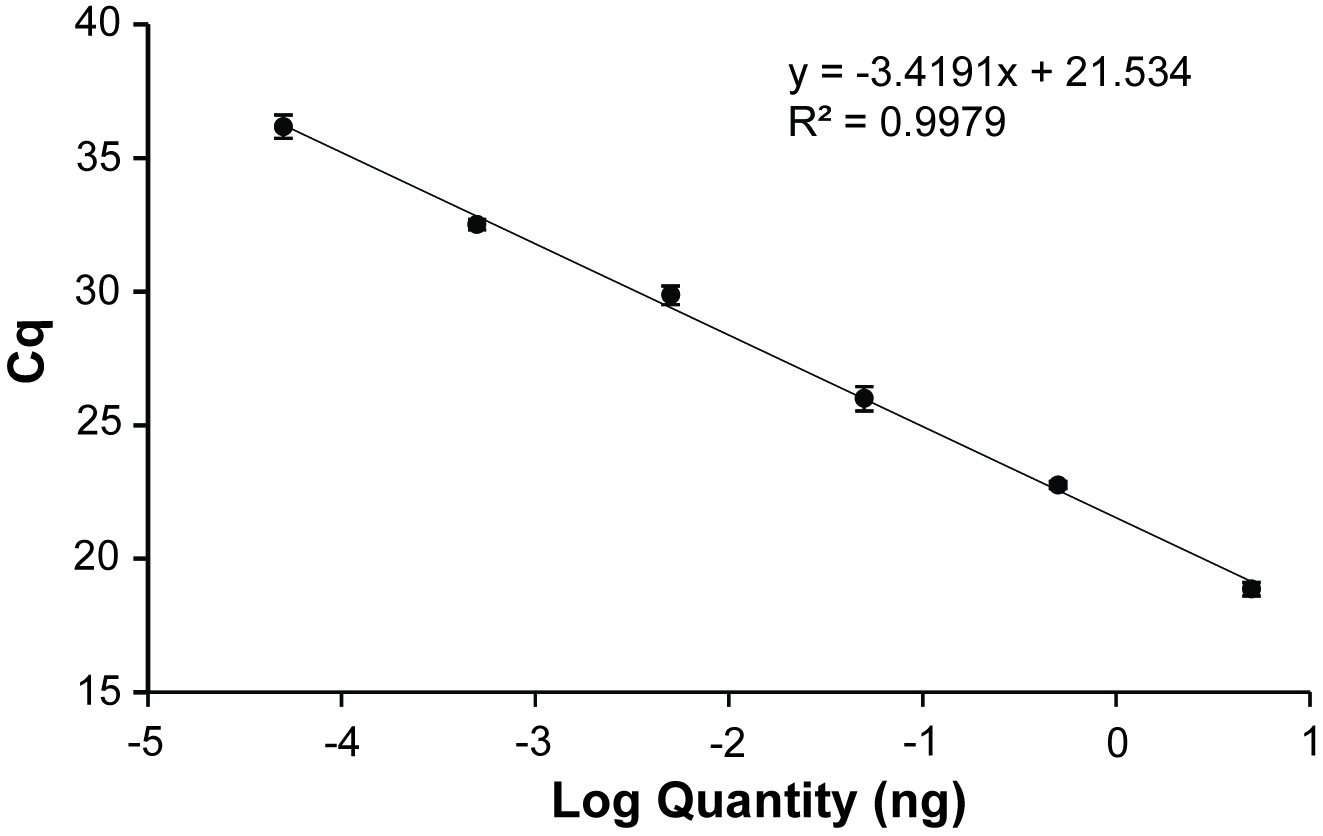


**Fig. S3** Melting curve of qPCR amplifying unknown samples, showing positive amplification of golden mussel but skewed toward NTC. Cq 33, 34, and 35 were assigned to amplifications similar with A, B, and C, respectively.


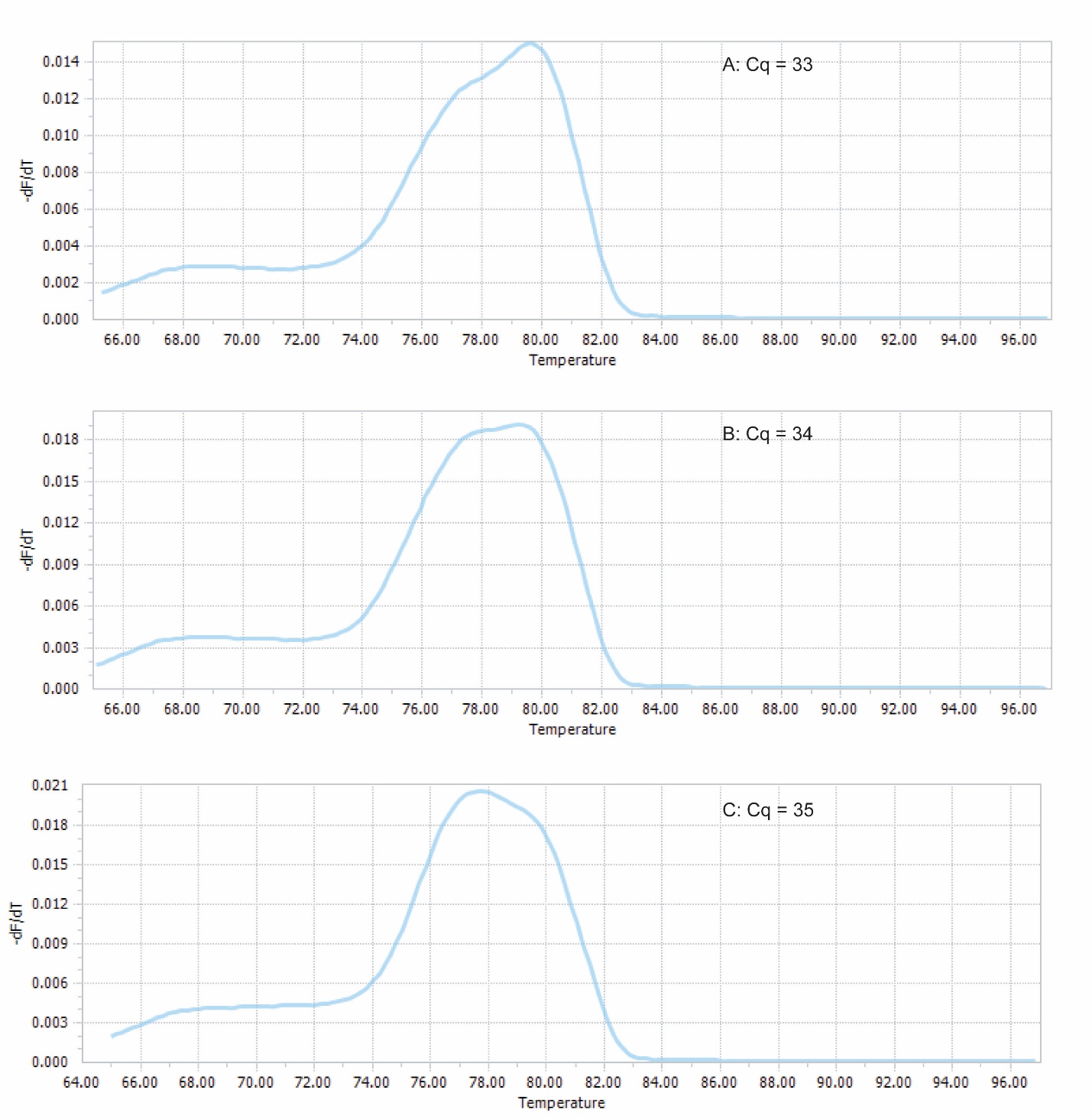

Supplement: Supplementary file 1 [file ECE3-8-11799-s001.docx]
